# Bone and Joint Infections in Tropical Settings: High Prevalence of Gram-Negative Bacilli and Implications for Empirical Therapy

**DOI:** 10.1093/ofid/ofag024

**Published:** 2026-01-15

**Authors:** Carla Pizzinat, Sylvaine Bastian, Frédéric Desmoulins, Elodie Curlier, Sébastien Breurec, Olivier Lesens, Kinda Schepers, Samuel Markowicz, Julien Coussement, Tanguy Dequidt

**Affiliations:** Department of Infectious Diseases, University Hospital of Guadeloupe, Les Abymes, Guadeloupe, France; Laboratory of Clinical Microbiology, University Hospital of Guadeloupe, Les Abymes, Guadeloupe, France; PCCEI, University of Montpellier, INSERM, EFS, Montpellier, France; Department of Trauma and Orthopaedic Surgery, University Hospital of Guadeloupe, Les Abymes, Guadeloupe, France; Department of Infectious Diseases, University Hospital of Guadeloupe, Les Abymes, Guadeloupe, France; Laboratory of Clinical Microbiology, University Hospital of Guadeloupe, Les Abymes, Guadeloupe, France; PCCEI, University of Montpellier, INSERM, EFS, Montpellier, France; Transmission, Reservoir and Diversity of Pathogens Unit, Pasteur Institute of Guadeloupe, Pointe-à-Pitre, France; Faculty of Medicine Hyacinthe Bastaraud, University of the Antilles, Pointe-à-Pitre, France; Centre for Clinical Investigation 1424, INSERM, Pointe-à-Pitre/Les Abymes, France; Infectious and Tropical Diseases Unit, University Hospital of French Guiana, Cayenne, French Guiana, France; Department of Infectious Diseases, University Hospital of Guadeloupe, Les Abymes, Guadeloupe, France; Department of Infectious Diseases, University Hospital of Guadeloupe, Les Abymes, Guadeloupe, France; Department of Infectious Diseases, University Hospital of Guadeloupe, Les Abymes, Guadeloupe, France; Department of Infectious Diseases, University Hospital of Guadeloupe, Les Abymes, Guadeloupe, France; Faculty of Medicine Hyacinthe Bastaraud, University of the Antilles, Pointe-à-Pitre, France; Centre for Clinical Investigation 1424, INSERM, Pointe-à-Pitre/Les Abymes, France

**Keywords:** bone joint infections, cefazolin-resistant, empirical antibiotic management, gram-negative bacilli, tropical settings

## Abstract

**Background:**

Bone and joint infections (BJIs) are increasingly reported worldwide, but data on their epidemiology remain limited in tropical settings. We aimed to characterize the causative agents of BJIs and their resistance patterns, in order to inform empirical antibiotic in our tropical setting.

**Methods:**

This 6-year retrospective study included all adults with a first microbiologically confirmed episode of BJI between January 2019 and December 2024 at the Guadeloupe University Hospital, a tertiary care center in the Caribbean.

**Results:**

A total of 312 patients with BJI were included. Among the 449 isolates recovered, Gram-negative bacilli (GNB) were predominant (41%), including AmpC β-lactamase–producing Enterobacterales (13%, 59/449) and *Pseudomonas aeruginosa* (9%, 39/449). Methicillin-resistant *Staphylococcus aureus* accounted for 3% (13/449). At least one GNB was identified in 31% of native septic arthritis (27/88), 33% of spondylodiscitis (9/27), 38% of prosthetic joint infections (27/71), 47% of osteosynthesis-associated infections (48/103), and 52% of osteomyelitis (12/23). Factors independently associated with GNB infection were a history of bite/scratch wound, contact with soil/vegetation and lower limb infection. Cefazolin provided limited likelihood of *in vitro* adequacy against causal pathogens in native septic arthritis episodes (74%, as compared to 92% for both cefepime and piperacillin-tazobactam). Lower rates were observed in cases of osteosynthesis-associated and prosthetic joint infections (48%–68%, as compared to 62%–75% for third-generation cephalosporins, 79%–80% for cefepime and 80%–86% for piperacillin–tazobactam).

**Conclusions:**

Our findings highlight the prominent role of GNBs in tropical BJI and support the implementation of local surveillance systems to guide empirical treatment strategies.

Bone and joint infections (BJIs) represent a growing public health concern due to their increasing incidence and considerable economic burden [1, 2]. BJIs include a wide spectrum of infections, such as native septic arthritis, osteomyelitis, septic pseudarthrosis, spondylodiscitis, prosthetic joint infections, and osteosynthesis-associated infections. Managing patients suffering from these infections requires a multidisciplinary approach and effective antibiotic therapy to prevent functional impairments [3].

In France, empirical antibiotic therapy (ie, antibiotic initiated before culture and susceptibility results are available) for BJI follows the official recommendations of the Société de Pathologie Infectieuse de Langue Française (SPILF), which are based on mainland bacterial epidemiological data [4–6]. However, their applicability to overseas territories like French West Indies is limited, given the distinct pathogen profiles and antibiotic resistance patterns observed in these regions [7, 8]. In Guadeloupe, previous hospital-based studies have highlighted specific features, including a high prevalence of AmpC β-lactamase–producing Enterobacterales—such as the *Enterobacter cloacae* complex— as well as extended-spectrum β-lactamase (ESBL)–producing Enterobacterales in clinical isolates from inpatients [9, 10]. Similarly, recent epidemiological studies from the Caribbean area and Latin America have reported a high incidence of Gram-negative bacilli (GNB) infections, particularly in intensive care units. These elements underscore region-specific bacterial profiles in terms of both species composition and resistance patterns [11]. Notably, GNB infections are often associated with antimicrobial resistance, which potentially complicates antibiotic management [12]. Such epidemiological specificities challenge the applicability of French national guidelines to tropical settings like the French West Indies. We hypothesized that GNB are more prevalent in tropical BJIs than in temperate regions. In Guadeloupe, where no data are currently available on the microbial epidemiology of BJIs—and more broadly in tropical regions, where such studies remain scarce—this knowledge gap represents a significant obstacle to the development of appropriate empirical treatment strategies. The aim of this study was therefore to characterize the microorganisms associated with BJIs and their resistance profiles at our tertiary institution located in a tropical area.

## MATERIALS AND METHODS

### Study Design and Population

We conducted a retrospective, observational, single-site study between January 2019 and December 2024 at the University Hospital of Guadeloupe, a tertiary care center in the French West Indies.

Eligible patients were adults (≥ 18 years) with a first microbiologically confirmed episode of BJI. Exclusion criteria included: isolated skin/soft tissue infections (ie, without BJI), diabetic foot infections, amputation stump infections, postoperative spinal infections, osteomyelitis associated with pressure ulcers, osteomyelitis associated with open fracture without implants, and osteomyelitis of the skull, sternum, or ribs. Other exclusion criteria were non-consent to use of medical data, and lack of access to clinical records.

### Definitions

We divided BJIs into (1) native septic arthritis, (2) spondylodiscitis, (3) osteomyelitis, (4) prosthetic joint infections, and (5) osteosynthesis-associated infections. Native septic arthritis was defined as the association of a positive joint fluid culture with clinical signs of local inflammation. Spondylodiscitis was defined as the association of a positive culture from a disco-vertebral or blood sample with both spinal pain and suggestive radiological abnormalities. Osteomyelitis was defined as the association of a positive bone, deep tissue and/or blood culture with suggestive clinical and radiological abnormalities. Osteomyelitis included episodes acquired through hematogenous dissemination, contiguous spread, and direct inoculation, as well as episodes of septic pseudarthrosis (defined as absence of bone consolidation after twice the expected healing time, in the absence of bone implant). Prosthetic joint infections or osteosynthesis-associated infections were defined as the association of a positive culture from a deep tissue or blood sample with suggestive clinical signs.

A positive culture was defined as either at least one positive culture showing a typical pathogen (eg, *Staphylococcus aureus*, *Escherichia coli*) or at least two positive cultures with a low-virulence organism (eg, coagulase-negative staphylococci [CoNS], *Corynebacterium* species, or *Cutibacterium acnes*). Polymicrobial BJIs were defined as those with at least two different organisms.

### Data Collection

Eligible patients were identified through both (1) systematic and comprehensive review of local data from our Microbiology laboratory and (2) coding data, using International Classification of Diseases, 10th Revision (ICD-10) codes retrieved from the hospital information system (see Supplementary Table 1 for ICD-10 codes used for screening).

Data were manually extracted from patient medical records and double-checked by two study investigators (CP and TD). Discrepancies were reviewed jointly and resolved by consensus. Because discrepancies were rare (<5%), inter-rater reliability was not formally assessed. We extracted demographic data (age, sex) and Charlson comorbidity index [13]. Immunocompromising status was defined as the presence of at least one of the following (1) ongoing chemotherapy or other immunosuppressive therapy within 30 days prior to infection onset, including use of prolonged corticosteroid therapy defined as ≥10 mg/day of prednisone or equivalent for more than 30 consecutive days, and/or (2) history of solid organ or hematopoietic cell transplantation. Clinical (type, site, fever, pain, local inflammation, wound dehiscence, sinus tract) and biological characteristics (leukocytes and neutrophils counts, CRP level) of BJI were recorded. The date of diagnosis of BJI corresponded to the collection date of the first/initial clinical specimen from which a pathogen was identified. Healthcare-associated infections were defined as recent exposure to healthcare settings, including intravenous therapy, wound or nursing care, hemodialysis, or chemotherapy within the past 30 days; hospitalization ≥48 hours prior to infection; residence in a nursing home or long-term care facility within the past 90 days; or infection within one year of prosthesis or osteosynthesis material placement. All other infections were considered as community-acquired. For implant-associated infections (ie, prosthetic joint infections, and osteosynthesis-associated infections), we recorded the date of implantation, type of implant, and time between implantation and infection. When applicable, specific mechanisms of injury were collected, including (human, animal, insect) bites/scratches, gunshot or stab wounds, road traffic accidents, and contact with soil or vegetation.

### Primary and Secondary Objectives

The primary study objective was to describe the clinical and microbial epidemiology of BJI in our tropical setting. Secondary objectives were (1) to describe the distribution of pathogens according to the type of BJI; (2) to measure the frequency of antibiotic-resistant bacteria; (3) to identify factors associated with GNB-related infections; and (4) to evaluate the adequacy of empirical antibiotic regimen which are commonly recommended in France.

### Microbiological Samples and Antimicrobial Susceptibility Testing

All strains were isolated from local or blood samples collected in a clinical context, either during surgery or at the patient's bedside. When appropriate, samples were mechanically homogenized and plated on various agar media (Columbia blood agar with 5% sheep blood, chocolate agar, bromophenol blue agar plates), and Schaedler enrichment broth (BioMerieux, Marcy L’Etoile, France). Cultures were incubated at 37°C under 5% CO_2_ and anaerobic conditions for ten days. All clinical isolates were identified by matrix-assisted laser desorption ionization-time of flight mass spectrometry (MALDI-TOF MS, VITEK® MS system, bioMérieux, Marcy l’Etoile, France), according to the manufacturer's instructions. Antibiotic susceptibility testing was performed using the disk diffusion method on Mueller–Hinton agar and interpreted according to the 2024 guidelines of the Antibiogram Committee of the French Society of Microbiology—European Committee on Antimicrobial Susceptibility testing (CA-SFM-EUCAST). Enterobacterales isolates showing resistance to at least one third-generation cephalosporin were classified as ESBL-producing based on a positive double-disk synergy test, in accordance with CA-SFM/EUCAST guidelines.

### *In Vitro* Adequacy of Empirical Antibiotics

We assessed the potential adequacy of main antibiotics (cefazolin, third-generation cephalosporins, cefepime, piperacillin-tazobactam, and carbapenems) and their possible associations with a second agent (vancomycin, daptomycin, or amikacin) based on susceptibility testing results. Notably, cefazolin is the SPILF-recommended empiric antibiotic for native joint septic arthritis when empiric therapy is required, which motivated its inclusion in this analysis [5]. For the purpose of this study, (1) methicillin-susceptible staphylococci were considered susceptible to other main beta-lactams (ie, third-generation cephalosporins, cefepime, piperacillin-tazobactam, and carbapenems); (2) AmpC-producing Enterobacterales were defined according to IDSA guidance [14] and included *Enterobacter cloacae* complex*, Klebsiella aerogenes, Citrobacter freundii* complex*, Serratia marcescens, Morganella morganii* and *Providencia spp.*; these isolates were considered susceptible to third-generation cephalosporins when susceptibility was observed on antibiotic susceptibility testing; (3) enterococci were considered not covered by piperacillin–tazobactam and carbapenems, as susceptibility to these agents was not routinely tested in our laboratory. In case of missing data (ie, when an agent *in vitro* activity was not tested), interpretation was based on the most frequently described spectrum of activity in the literature, as detailed in Supplementary Table 2.

### Statistical Analysis

Continuous variables were expressed as medians [interquartile ranges] and compared using the Student's *t*-test or the Mann-Whitney *U* test, depending on data distribution. Categorical variables were expressed as counts and percentages and compared using the Chi-square test or Fisher's exact test, as appropriate. The number of missing data was reported for each variable. We performed a multivariate logistic regression analysis using backward selection to identify factors associated with GNB-related infection, including variables with *P* < .10 in univariate analysis and retaining those with *P* < .10 in the final model. Multicollinearity was assessed using variance inflation factor (VIF), and all variables exhibited low VIF values (<2), indicating no evidence of multicollinearity. Model fit was acceptable according to the Hosmer–Lemeshow test (χ² = 7.39, *P* = .12). All tests were two-tailed, and a *P*-value ≤ .05 was considered statistically significant. Data were analyzed using STATA® software (version 18, StataCorp, College Station, TX, USA).

### Ethics

This study was approved by the national ethics committee for infectious diseases (Société de Pathologie Infectieuse de Langue Française, SPILF; approval number 2024–0506-2). In accordance with French legislation, participants received an information notice detailing their participation in the study and offering them the opportunity to object. Following the guidelines of the French Commission Nationale de l’Informatique et des Libertés (CNIL), all data were pseudonymized by assigning an identification number to each patient upon inclusion.

## RESULTS

### Patient Characteristics

**Figure 1.**
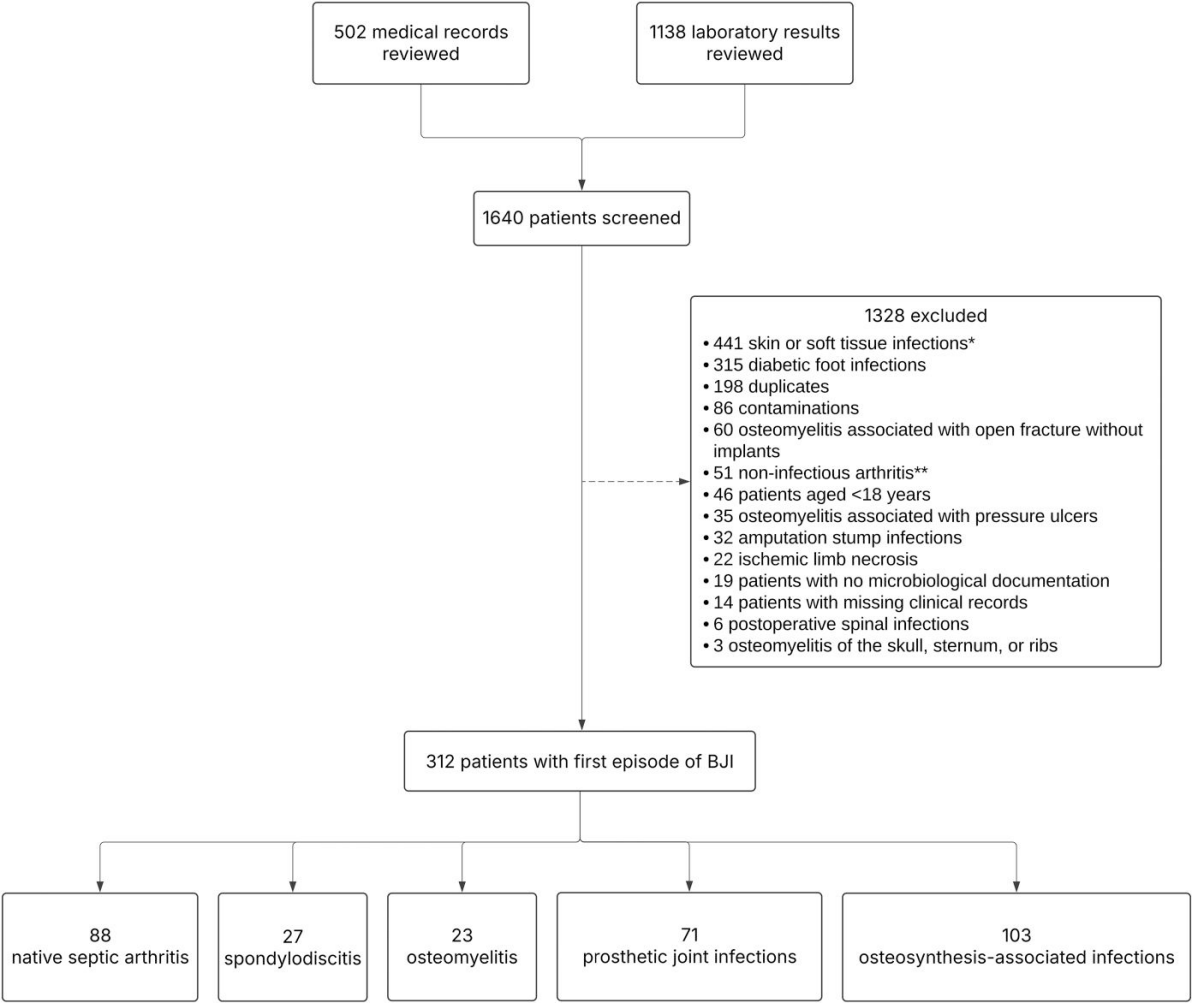
Flow chart. BJI, Bone and joint infections. *Skin or soft tissue infections included abscess (*n* = 224), necrotizing or non-necrotizing cellulitis (*n* = 118), phlegmons (*n* = 60), felons (*n* = 33), bursitis (*n* = 3), tenosynovitis (*n* = 2), and tumors with secondary infection (*n* = 1). **Non-infectious arthritis included crystal-induced arthritis (*n* = 29), degenerative arthritis (*n* = 15), and inflammatory arthritis (*n* = 7).

We included 312 patients with a first episode of BJI over the 6-year study period (2019–2024), after screening medical records from 1640 patients (see flow chart in Figure 1). The most common type of BJI was osteosynthesis-associated infections (33%, 103/312), followed by native septic arthritis (28%, 88/312), prosthetic joint infections (23%, 71/312), spondylodiscitis (9%, 27/312), and osteomyelitis (7%, 23/312).

Characteristics of patients and infections are presented in Table 1. Most infections were monomicrobial (76%, 236/312) and just over half of them were healthcare-associated (53%, 166/312). The origin of arthritis was hematogenous (41%, 36/88), direct inoculation (34%, 30/88), contiguous spread (19%, 17/88), or unknown (6%, 5/88). The origin of spondylodiscitis was hematogenous (85%, 23/27) or unknown (15%, 4/27). For device-associated infections, the median time between implantation and infection was 37 days [18–149] for osteosynthesis-associated infections and 287 [26–2096] days for prosthetic joint infections. These were classified as acute postoperative (≤ 3 months) (45%, 32/71), delayed/chronic (38%, 27/71), or acute hematogenous (17%, 12/71).

**Table 1.** Baseline characteristics

Characteristics	Total (*n* = 312^a^)	Native Septic Arthritis (*n* = 88)	Spondylodiscitis (*n* = 27)	Osteomyelitis^b^ (*n* = 23)	Prosthetic Joint Infections (*n* = 71)	Osteosynthesis-associated Infections (*n* = 103)
Age, *years*	60 [39–73]	56 [36–70]	66 [59–75]	47 [37–57]	72 [63–81]	47 [32–66]
Male	207 (66)	64 (73)	15 (56)	21 (91)	41 (58)	66 (64)
Charlson index	2 [0–5]	2 [0–5]	4 [3–6]	0 [0–2]	4 [2–6]	0 [0–2]
Immunosuppression	18 (6)	9 (10)	7 (26)	0 (0)	1 (1)	1 (1)
Origin of infection						
Community-acquired	146 (47)	67 (76)	16 (60)	19 (83)	29 (40)	16 (15)
Healthcare-associated	166 (53)	21 (24)	11 (40)	4 (17)	43 (60)	87 (85)
Presenting signs						
Fever	106 (34)	52 (59)	23 (85)	2 (9)	17 (24)	12 (12)
Pain	236 (76)	85 (97)	26 (96)	16 (67)	48 (67)	61 (59)
Local inflammation	228 (73)	83 (94)	0 (0)	19 (83)	47 (65)	80 (78)
Wound dehiscence	115 (37)	8 (9)	0 (0)	5 (22)	24 (32)	79 (77)
Sinus tract	30 (10)	5 (6)	0 (0)	3 (13)	7 (10)	15 (15)
Site of infection^c^						
Upper limb	54 (17)	24 (27)	0 (0)	9 (39)	1 (1)	20 (19)
Lower limb	229 (73)	62 (71)	0 (0)	14 (61)	70 (99)	83 (81)
Spine	27 (9)	0 (0)	27 (100)	0 (0)	0 (0)	0 (0)
Multiple sites (≥2)	2 (1)	2 (2)	0 (0)	0 (0)	0 (0)	0 (0)
Events prior to infection						
Bite	14 (5)	11 (12)	0 (0)	3 (12)	1 (1)	1 (1)
Gunshot wound	6 (2)	1 (1)	0 (0)	2 (8)	2 (2)	1 (1)
Stab wound	6 (2)	3 (3)	0 (0)	1 (4)	2 (2)	0 (0)
Road traffic accident	34 (11)	6 (7)	0 (0)	1 (4)	27 (26)	1 (1)
Contact with soil/plants	31 (10)	8 (9)	0 (0)	4 (15)	19 (18)	0 (0)
Biological parameters						
Leukocytes, *Giga/l*	9.8 [6.9–13.4]	12.4 [9.2–16]	9.8 [6.4–13.7]	9.3 [6.6–11.7]	8.6 [6.8–11.7]	9.5 [6.4–12.1]
Neutrophils, *Giga/l*	7.1 [4.6–10.7]	9.4 [5.8–12.9]	6.6 [5.2–9]	5.1 [3.2–9.6]	6.4 [4.7–8.9]	6.9 [4.4–9.6]
CRP, *mg/l*	109 [42–216]	212 [84–328]	116 [42–220]	22 [9.2–120]	89 [49.2–172]	85 [20–153]
Blood cultures	170 (54)	62 (70)	25 (93)	8 (35)	36 (51)	39 (38)
Positivity rate	69 (22)	28 (32)	17 (68)	2 (9)	17 (24)	5 (13)
Number of local samples	2 [1–3]	1 [1–2]	1 [0–2]	3 [2–4]	3 [2–4]	3 [1–3]
Polymicrobial infection	76 (24)	17 (19)	1 (4)	6 (26)	12 (17)	40 (39)

Data are presented as median [IQR] or count (%). CRP, C-reactive protein; NA, Not applicable. Missing data concerned leukocytes (*n* = 13), neutrophils (*n* = 18), and CRP (*n* = 16).

^a^Ten patients presented with two BJI conditions and, to avoid duplicates in data analysis, were included in only a single subgroup after medical history review (details are presented in Supplementary Appendix p. 5).

^b^Osteomyelitis encompasses cases resulting from hematogenous spread (*n* = 2), contiguous extension (*n* = 14), direct inoculation (*n* = 3) or unknown (*n* = 1), as well as septic pseudarthrosis (*n* = 3).

^c^Further details on the sites of infection are described in Supplementary Appendix (p. 5).

### Microbiology and Antimicrobial Resistance

A total of 449 organisms were identified from 312 patients with BJI. The full list of microorganisms identified is available in Supplementary Table 3. The most common pathogens were GNBs (41%, 182/449), including significant proportions of both AmpC β-lactamase–producing Enterobacterales (13%, 59/449) and *Pseudomonas aeruginosa* (9%, 39/449). Only 2% of all pathogens were ESBL-producing Enterobacterales (8/449). Staphylococci represented 36% (160/449) of all pathogens (with *S. aureus* being more common than CoNS, 30% [132/449] vs 6% [28/449]); 92% of all staphylococci were susceptible to methicillin (147/160), 12.5% displayed macrolide-lincosamide-streptogramin B (MLSB) inducible resistance (20/160). Streptococci accounted for 71/449 isolates (16%), with *Streptococcus agalactiae* being the most common species (5%, 24/449).

**Figure 2.**
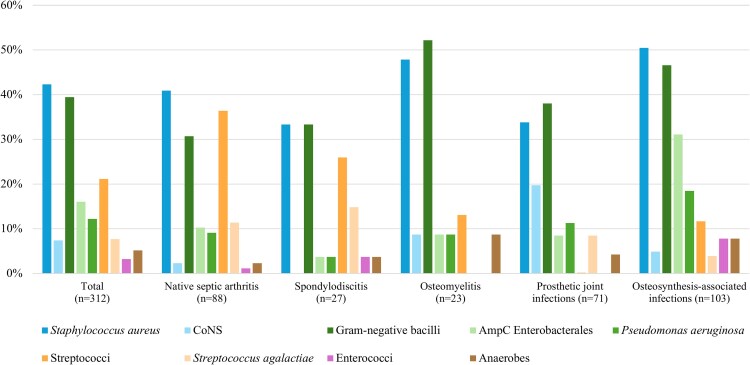
Proportion of patients (%) with at least one isolate from each microbial group, stratified by type of bone and joint infection (BJI). CoNS, Coagulase-negative staphylococci; AmpC Enterobacterales, AmpC β-lactamase–producing Enterobacterales. Bars are not mutually exclusive. GNB included *Pseudomonas aeruginosa*, AmpC β-lactamase–producing Enterobacterales, and anaerobic GNB; Streptococci included *Streptococcus agalactiae*. Proportions and percentages of the microbial groups are detailed in Supplementary Appendix (p. 6).

Figure 2 shows the proportion of patients with at least one isolate from each microbial group. Overall, a GNB was identified in at least 30% of all types of BJIs: 52% (12/23) in osteomyelitis, 47% (48/103) in osteosynthesis-associated infections, 38% (27/71) in prosthetic joint infections, 33% (9/27) in spondylodiscitis and 31% (27/88) in native septic arthritis. Proportions for the remaining microbial groups are detailed in Supplementary Appendix (p. 6).

### Factors Associated With GNB-Related Infection

In univariate analysis, factors significantly associated with the identification of a GNB were contact with soil/vegetation, a history of road traffic accident, lower limb localization, and bite/scratch wound (see Table 2). In multivariate analysis, bite/scratch wound (OR 12.51; 95% CI 3.17–49.30; *P* < .001), contact with soil/vegetation (OR 6.36; 95% CI 2.55–15.92; *P* < .001), and lower limb localization (OR 3.98; 95% CI 1.71–9.26; *P* = .001) remained independently associated with GNB-related infection.

**Table 2.** Factors Associated With Gram-negative Bacilli Detection in Bone and Joint Infections

Characteristics	GNB *n* = 123	Non-GNB *n* = 189	Univariate Analysis		Multivariate Analysis	
			OR (95%CI)	*P* value	aOR (95%CI)	*P* value
Age, *years*	56 [35–73]	61 [45–73]	0.99 (.98–1.00)	.141		
Male	78 (63)	129 (68)	1.24 (.75–2.05)	.377		
Charlson	2 [0–5]	2 [0–5]	0.94 (.87–1.03)	.180		
Immunosuppression	5 (6)	11 (6)	0.98 (.31–2.85)	.962		
Origin						
Community-acquired	47 (38)	99 (52)	Reference variable			
Healthcare-associated	76 (62)	90 (48)	1.78 (1.09–2.90)	.**014**	1.64 (.96–2.80)	.072
Site						
Upper limb	12 (11)	42 (22)	Reference variable			
Lower limb	101 (82)	128 (68)	2.19 (1.22–4.00)	**.005**	3.98 (1.71–9.26)	.**001**
Spine	9 (7)	18 (10)	0.75 (.29–1.83)	.498		
Road traffic accident	22 (18)	12 (6)	3.21 (1.45–7.42)	.**001**		
Contact with soil or vegetation	23 (19)	8 (4)	5.20 (2.14–13.90)	**<**.**001**	6.36 (2.55–15.92)	**<**.**001**
Bite/scratch wound	10 (8)	4 (2)	4.09 (1.14–18.22)	.**022**	12.51 (3.17–49.30)	**<**.**001**
Gunshot wound	3 (2)	3 (2)	1.55 (.20–11.75)	.684		
Stab wound	0 (0)	6 (3)	NA	.085		

Data are presented as median [IQR] or count (%). *P* values in bold are statistically significant.

aOR, adjusted odds ratio; GNB, Gram-negative bacilli; NA, Not applicable; OR, Odds ratio. No data were missing.

### Estimated Adequacy of Empirical Antibiotic Regimens

**Figure 3.**
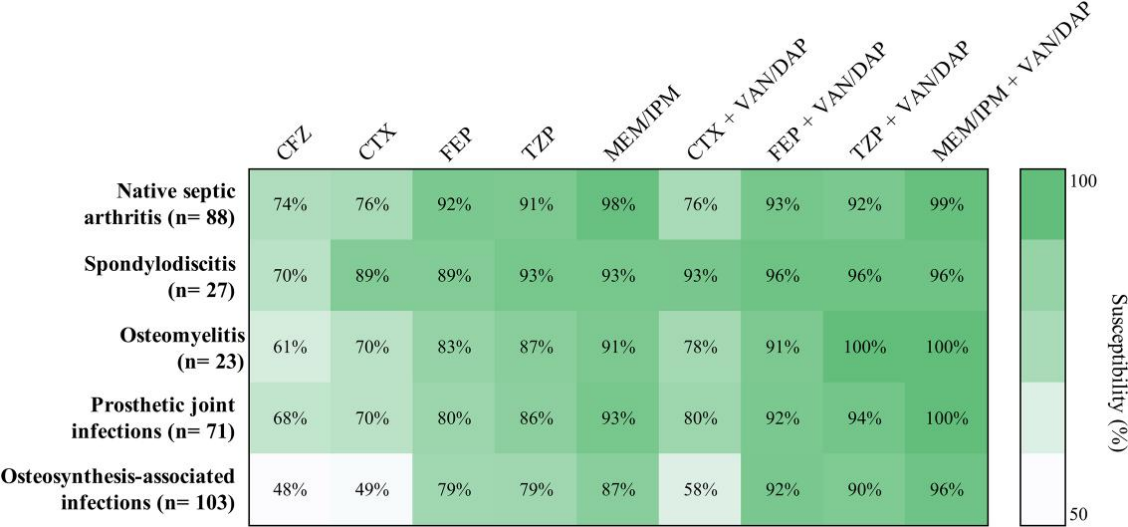
Susceptibility (%) heatmap of antibiotic combinations in BJIs. AMK, amikacin; CFZ, cefazolin; CTX, cefotaxime or ceftriaxone; DAP, daptomycin; FEP, cefepime; IMI, imipenem; MEM, meropenem; VAN, vancomycin; TZP, piperacillin-tazobactam.

The potential adequacy of main empirical antibiotic regimens stratified by infection type is presented in Figure 3. Cefazolin demonstrated < 75% *in vitro* adequacy against causative pathogens in all five types of infection, including native septic arthritis (74%, 65/88) and spondylodiscitis (70%, 19/27). Susceptibility rates for cefepime and piperacillin-tazobactam were higher, exceeding 79% across all categories. Among the 23 patients with native septic arthritis due to cefazolin-resistant bacteria (see Table 3), the most frequently identified bacteria were *Pseudomonas aeruginosa* (35%, 8/23), AmpC β-lactamase–producing Enterobacterales (35%, 8/23), *Aeromonas* spp (9%, 2/23), and *Pasteurella* spp (9%, 2/23).

**Table 3.** Characteristics of the 23 Patients With Native Septic Arthritis Due to Cefazolin-resistant Bacteria

…	Age, *Years*	Sex	Charlson	Site	Immunocompromising Conditions	Healthcare-associated Infection	Road Traffic Accident	Bite Or Scratch Wound	Contact With Soil Or Vegetation	Gunshot Wound	Blood Culture Positives	Polymicrobial	Cefazolin-resistant Bacteria
1	32	Male	0	Knee	No	No	Yes	No	Yes	No	No	Yes	*E. cloacae*
2	51	Female	3	Ankle	Yes	No	No	No	No	No	No	No	*P. aeruginosa*
3	67	Female	5	Knee	No	Yes	No	No	No	No	No	No	*P. aeruginosa*
4	45	Male	1	Hand	No	No	No	Yes	No	No	No	Yes	*P. aeruginosa*
5	38	Male	0	Elbow	No	No	No	Yes	No	No	No	Yes	*P. canis, Bacteroides* spp
6	30	Male	0	Foot	No	No	No	No	No	Yes	No	Yes	*P. aeruginosa*
7	41	Male	0	Knee	No	Yes	Yes	No	Yes	No	No	Yes	*E. cloacae*
8	35	Female	0	Hand	No	No	No	No	No	No	No	No	*P. aeruginosa*
9	70	Male	9	Hip	Yes	Yes	No	No	No	No	No	No	*P. aeruginosa*
10	31	Male	0	Hip	No	Yes	No	No	No	No	No	Yes	*E. cloacae*
11	22	Male	0	Knee	No	Yes	Yes	No	Yes	No	No	Yes	*A. hydrophila, E. cloacae*
12	42	Male	0	Knee	No	No	No	Yes	No	No	No	Yes	*P. aeruginosa*
13	69	Female	4	Shoulder	Yes	Yes	No	No	No	No	No	No	*E. coli* ^a^
14	75	Male	4	Foot	No	No	No	No	Yes	No	No	Yes	*A. caviae*
15	31	Male	0	Knee	No	Yes	No	No	No	No	No	No	*S. marcescens*
16	30	Male	0	Elbow	No	Yes	Yes	No	Yes	No	No	Yes	*P. aeruginosa, E. cloacae*
17	46	Male	0	Knee	No	No	No	Yes	No	No	No	Yes	*E. cloacae*
18	49	Male	0	Knee	No	No	No	Yes	No	No	No	Yes	*Pasteurella* spp
19	29	Male	0	Knee	No	Yes	No	No	No	No	No	No	*E. cloacae*
20	55	Male	2	Knee	No	No	No	No	Yes	No	No	Yes	*E. cloacae*
21	46	Male	0	Knee, ankle	No	No	No	No	No	No	No	No	*N. gonorrhoeae*
22	82	Female	12	Ankle	Yes	Yes	No	No	No	No	No	No	*K. pneumoniae* ^b^
23	54	Male	5	Hip	Yes	No	No	No	No	No	No	No	*M. genavense*

*E.cloacae* = *Enterobacter cloacae*. *P. aeruginosa* = *Pseudomonas aeruginosa*. *P. canis* = *Pasteurella canis. A.hydrophila* = *Aeromonas hydrophila. E.coli* = *Escherichia coli. A. caviae* = *Aeromonas caviae. S. marsescens* = *Serratia marcescens.*

*N. gonorrhoeae* = *Neisseria gonorrhoeae. K.pneumoniae* = *Klebsiella pneumoniae. M. genavense* = *Mycobacterium genavense.*

^a^High-level penicillinase-producing *Escherichia coli*.

^b^ESBL-producing *Klebsiella pneumoniae*.

Pathogens isolated from prosthetic joint infections showed higher susceptibility rates to cefepime and piperacillin–tazobactam (80% and 86%, respectively) than to cefazolin and third-generation cephalosporins (68% and 70%), with a similar trend observed among isolates from osteosynthesis-associated infections (79% both vs 48% and 49%). Combinations of cefepime or piperacillin-tazobactam with glycopeptides showed higher *in vitro* susceptibility rates ≥ 90% in every infection group.

## DISCUSSION

To the best of our knowledge, this study is the first to comprehensively characterize the microbial epidemiology of distinct clinical subtypes of BJI in a tropical setting. One of its key findings is the consistently elevated proportion of GNBs across the various subtypes of BJI. Specifically, GNBs were present in more than 30% of patients in each subgroup, well above the 17%–25% range reported in earlier French series [6, 15, 16]. Observed proportions of GNBs were even lower in other European studies, ranging from 6% to 19% [2, 17]. Furthermore, a substantial proportion of GNB infections were associated with “difficult-to-treat” pathogens, including to AmpC β-lactamase–producing Enterobacterales and *Pseudomonas aeruginosa*, which accounted for with 16% and 12% of all infections, respectively. By comparison, a recent study from southern France reported lower proportions of these two pathogens (7% and 5%, respectively), and a Spanish study found even lower rates (<1% and 2%, respectively) [2,16] .

When stratifying BJIs by different clinical subtypes, GNB accounted for 33% of spondylodiscitis cases, a proportion close to that reported in a national German registry-based study (around 30%) [18], but higher than in other epidemiological studies (7–18%) [19, 20]. *E. coli* (19%) was the predominant species in our study, consistent with findings by Kehrer *et al* [21]. In native septic arthritis, GNB were identified in 31% of patients, a notably higher proportion than those reported in international studies (6–23%) [22–31]. Data from other tropical regions remain scarce but suggest a lower GNB involvement: Enterobacterales accounted for 11% of cases in Mali [32], and GNB for 11% in Thailand (excluding *Burkholderia pseudomallei*, which represented 48% of cases due to its region-specific epidemiology) [33].

Given the high prevalence of GNB in our cohort, we subsequently investigated factors associated with their identification in patients with BJIs. The strongest independent factor was a history of bite or scratch wounds, consistent with previous reports describing *Pasteurella* spp. and other oral commensals from humans or animals as causative agents of BJIs [34]. Although less frequently reported, environmental exposure—particularly contact with soil or vegetation—was also significantly associated with the identification of a GNB. In our study, several species of *Aeromonas* (ie, *A. caviae*, *A. hydrophila*, and *A. jandaei*) were identified, accounting for 2% of all isolates. Although uncommon, *Aeromonas* spp. intrinsically resistant to cefazolin has been implicated in BJIs, particularly following water-related trauma in tropical environments [35]. Thirdly, we also found lower limb localization to be associated with GNB, a finding previously reported in the literature [17]. Finally, healthcare-associated infections were significantly associated with GNB identification in univariate analysis but this association did not reach statistical significance in multivariate analysis (*P* = .07). Although such infections have previously been identified as a risk factor for GNB (28), GNBs were not among the three most common causative agents in a case series of iatrogenic native joint infections [36], suggesting variability depending on the clinical context and study population. The reasons underlying the high prevalence of GNB infections in BJIs in our study remain unclear. However, several environmental factors may contribute to this distinct epidemiological pattern. Elevated ambient temperatures, as commonly observed in tropical climates, can accelerate bacterial growth and enhance the expression of virulence factors, rendering certain pathogens more aggressive or more prone to cause infection [37]. In parallel, sustained high humidity may promote prolonged skin moisture, potentially compromising the integrity of the cutaneous barrier and facilitating bacterial translocation into underlying tissues. Together, these environmental conditions may promote the pathogenicity of GNB in tropical settings. Nonetheless, this explanation remains hypothesis-generating, as no robust evidence directly links tropical environmental factors to BJI microbiology. Further studies are needed to confirm these hypotheses and to better delineate the respective contributions of climatic and environmental factors to the epidemiology of BJIs in such regions.

The SPILF guidelines recommend empirical antibiotic therapy with cefazolin (or penicillin M) for native joint septic arthritis associated with sepsis, or when direct examination is negative despite purulent synovial fluid. However, in our study, the estimated efficacy of cefazolin was only 74%, compared with 92% for cefepime and 91% for piperacillin-tazobactam. After reviewing cases involving cefazolin-resistant pathogens, we found that most patients presented with one or more of the previously identified risk factors: bite or scratch wounds, contact with soil or vegetation, or lower limb localization. Therefore, we suggest that clinicians should systematically assess the presence of these risk factors in order to guide empirical therapy [5].

Bacterial isolates from prosthetic joint infections also showed higher susceptibility rates to cefepime (80%) and piperacillin–tazobactam (86%) than to cefazolin (68%) and third-generation cephalosporins (70%). These findings are consistent with those of Vidal *et al*., who reported a reduced adequacy of empirical therapy with third-generation cephalosporins (78%) compared with piperacillin–tazobactam (90%) in microbiologically confirmed BJIs [38]. The high proportion of GNB (38%) in our study, including *Pseudomonas aeruginosa* (11%) and AmpC β-lactamase–producing Enterobacterales (8%), may explain the reduced susceptibility rates observed. These rates remain substantially higher than those generally reported in international studies (7–18%) [39–43], with the notable exception of an Australian study that reported a similarly high GNB prevalence (42%) [44]. Although the prevalence of methicillin-resistant staphylococci in our cohort was relatively low (7%) compared with rates reported in North America (up to 50%), the highest overall susceptibility rate (≥90%) was observed with the addition of a glycopeptide. This effect may be attributed not only to its activity against the few methicillin-resistant staphylococci, but also to its coverage of other bacteria such as enterococci, anaerobes, and *Bacillus cereus*. The benefits and harms of systematically covering these low-virulence pathogens using dual therapy remain to be determined.

Interestingly, in our study, *Streptococcus agalactiae* was the most frequently isolated streptococcal species, accounting for 11% of native septic arthritis cases and 15% of spondylodiscitis cases. These findings underscore the emerging importance of this organism as a significant pathogen in BJI in non-pregnant adults, expanding its traditionally recognized role beyond neonatal and obstetric infections [45].

Our study has several limitations. First, its retrospective, observational, single-center design inherently carries a risk of information bias, including incomplete or inaccurate data. To limit this risk, all medical records in our study were independently reviewed and cross-validated by two investigators. Second, our sample size is relatively limited; that said, to the best of our knowledge, our cohort represents the largest series on BJIs documented in a tropical setting, providing original and valuable data from a region for which epidemiological data are still lacking. Third, restricting the analysis to microbiologically confirmed BJIs may have introduced selection bias by excluding culture-negative cases. Fourth, osteomyelitis cases associated with open fractures without implants were excluded due to the retrospective inability to distinguish colonization from infection in acute cases in a significant proportion of them. Fifth, we were unable to assess patient outcomes due to the anticipated high rate of missing follow-up data. Sixth, the analysis of antibiotic susceptibility was based on *in vitro* data and therefore remains theoretical. In clinical practice, empirical antibiotic management is guided by multiple factors, including the patient's microbiological history and the severity of infection. Consequently, the rate of discordance between empirical antibiotic therapy and bacterial susceptibility was probably overestimated and is likely lower in real-life clinical practice.

In conclusion, our results revealed a particular epidemiological pattern in our tropical region, characterized by an unusually high proportion of GNB, which, to our knowledge, has never been observed elsewhere. Our study findings highlight the importance of continuous surveillance of local bacterial ecology to guide empirical antibiotic management and to inform antimicrobial stewardship strategies tailored to the distinctive microbiology of tropical regions. Further studies across tropical settings are needed to determine whether this high rate of GNB reflects a local specificity or a broader feature associated with tropical environments, and to compare the benefits and harms of different empirical approaches in such settings.

## Supplementary Material

ofag024_Supplementary_Data

## Supplementary Data

Supplementary materials are available at *Open Forum Infectious Diseases* online. Consisting of data provided by the authors to benefit the reader, the posted materials are not copyedited and are the sole responsibility of the authors, so questions or comments should be addressed to the corresponding author.
