# Supplementary material for: Bone and Joint Infections in Tropical Settings: High Prevalence of Gram-Negative Bacilli and Implications for Empirical Therapy
Source: Open Forum Infect Dis. 2026 Jan 15;13(2):ofag024. doi: 10.1093/ofid/ofag024 (PMC12877873; doi:10.1093/ofid/ofag024)
Supplement: ofag024_Supplementary_Data [file ofag024_supplementary_data.doc]

**Appendix**

**Supplementary Methods**

**Table S1 -** ICD-10 codes used to identify bone and joint infections

| **ICD-10 Code** | **Description** |
| --- | --- |
| M00 | Pyogenic arthritis |
| M00.0 | Staphylococcal arthritis and polyarthritis |
| M00.1 | Pneumococcal arthritis and polyarthritis |
| M00.2 | Other streptococcal arthritis and polyarthritis |
| M00.8 | Arthritis and polyarthritis due to other specified bacterial agents |
| M00.9 | Pyogenic arthritis, unspecified |
| M01.0 | Meningococcal arthritis |
| M01.1 | Tuberculous arthritis |
| M01.2 | Arthritis in Lyme diseases |
| M01.3 | Arthritis in other specified infectious and parasitic diseases |
| M01.6 | Arthritis in mycoses |
| M01.8 | Arthritis in other infectious and parasitic diseases classified elsewhere |
| M13.9 | Arthritis, unspecified |
| M96.8 | Other postprocedural musculoskeletal disorders |
| M46.2 | Osteomyelitis of vertebra |
| M46.3 | Infection of intervertebral disc (pyogenic) |

**Table S2 - Microbiological rules for susceptibility matching**

In cases where an antibiotic was not tested against a bacterial strain, its presumed activity was inferred from the most frequently reported spectrum of activity in the literature. The following organism-specific interpretations were used:

| **Bacterial species** | **Susceptible** | **Resistant** |
| --- | --- | --- |
| *Aeromonas spp.* | Cefepime, carbapenems | Cefazolin, G3C, piperacillin–tazobactam, vancomycin, daptomycin |
| *Bacillus cereus* | Carbapenems, vancomycin, daptomycin | Cefazolin, G3C, cefepime, piperacillin–tazobactam |
| *Bacteroides spp.* | Piperacillin–tazobactam, carbapenems | Cefazolin, G3C, cefepime, vancomycin, daptomycin |
| *Clostridium glycolycum* | Vancomycin, daptomycin | Cefazolin, G3C, cefepime, piperacillin–tazobactam |
| *Corynebacterium amycolatum* | Carbapenems, vancomycin, daptomycin | Cefazolin, G3C, cefepime, piperacillin–tazobactam |
| *Cutibacterium acnes* | Cefazolin, G3C, piperacillin–tazobactam, carbapenems, vancomycin, daptomycin | Cefepime |
| *Eikenella corrodens* | G3C, cefepime, piperacillin–tazobactam, carbapenems | Cefazolin, vancomycin, daptomycin |
| *Enterococcus avium* | Vancomycin, daptomycin | Cefazolin, 3GC, cefepime, piperacillin–tazobactam, carbapenems |
| *Enterococcus faecalis* | Vancomycin, daptomycin | Cefazolin, 3GC, cefepime, piperacillin–tazobactam, carbapenems |
| *Enterococcus faecium* | Vancomycin, daptomycin | Cefazolin, 3GC, cefepime, piperacillin–tazobactam, carbapenems |
| *Finegoldia magna* | Vancomycin, daptomycin, piperacillin–tazobactam, carbapenems | Cefazolin, G3C, cefepime |
| *Fusobacterium necrophorum spp* | G3C, cefepime, piperacillin–tazobactam, carbapenems | Cefazolin, vancomycin, daptomycin |
| *Klebsiella pneumoniae* | Cefazolin, G3C, cefepime, piperacillin–tazobactam, carbapenems | Vancomycin, daptomycin |
| *Myroides spp.* | / | All tested agents (pan-resistant) |
| *Prevotella spp.* | Piperacillin–tazobactam, carbapenems | Cefazolin, G3C, cefepim, vancomycin, daptomycin |
| *Parvimonas spp.* | Piperacillin–tazobactam, carbapenems, vancomycin, daptomycin | Cefazolin, G3C, cefepime |
| G3C=Third-generation cephalosporin | | |

**Supplementary Results**

**Table S3.** Classification of microbial isolates from bone and joint infections.

| **Microbial isolates** | **Total (n=449)** | **Arthritis (n=120)** | **Spondylodiscitis (n=28)** | **Osteomyelitis (n=34)** | **Periprosthetic joint**  **infections (n=89)** | **Osteosynthesis-associated infections (n=178)** |
| --- | --- | --- | --- | --- | --- | --- |
| **Gram-negative bacilli** |  |  |  |  |  |  |
| **Enterobacterales** |  |  |  |  |  |  |
| *Citrobacter koseri* | 1 | 0 | 0 | 0 | 1 | 0 |
| *Citrobacter freundii* | 1 | 0 | 0 | 0 | 0 | 1 |
| *Citrobacter werkmanii* | 2 | 0 | 0 | 0 | 0 | 2 |
| *Enterobacter cloacae* | 37 | 8 | 1 | 1 | 2 | 25 |
| *Enterobacter hormaechei* | 1 | 0 | 0 | 0 | 0 | 1 |
| *Escherichia coli* | 19 | 5 | 5 | 1 | 4 | 4 |
| *Escherichia hermanii* | 2 | 2 | 0 | 0 | 0 | 0 |
| *Klebsiella aerogenes*  *Klebsiella pneumoniae* | 5  22 | 0  6 | 0  1 | 1  3 | 0  6 | 4  6 |
| *Leclercia adecarboxylata* | 1 | 0 | 0 | 0 | 0 | 1 |
| *Morganella morganii* | 5 | 1 | 0 | 1 | 1 | 2 |
| *Proteus mirabilis* | 10 | 0 | 0 | 1 | 3 | 6 |
| *Providencia stuartii* | 1 | 0 | 0 | 0 | 0 | 1 |
| *Salmonella enterica* | 2 | 1 | 0 | 1 | 0 | 0 |
| *Serratia marcescens* | 6 | 1 | 0 | 0 | 3 | 2 |
| **Non-fermenting** |  |  |  |  |  |  |
| *Achromobacter xylosoxidans* | 1 | 0 | 0 | 0 | 0 | 1 |
| *Acinebacter baumannii* | 2 | 0 | 0 | 0 | 2 | 0 |
| *Myroides* sp | 1 | 0 | 0 | 0 | 0 | 1 |
| *Pseudomonas aeruginosa* | 39 | 8 | 1 | 2 | 9 | 19 |
| **HACEK group** |  |  |  |  |  |  |
| *Aggregatibacter actinomycetemcomitans* | 1 | 0 | 0 | 0 | 1 | 0 |
| *Aggregatibacter aphrophilus* | 1 | 0 | 1 | 0 | 0 | 0 |
| *Eikenella corrodens* | 2 | 0 | 0 | 2 | 0 | 0 |
| **Anaerobes** |  |  |  |  |  |  |
| *Bacteroides fragilis* | 2 | 0 | 0 | 1 | 1 | 0 |
| *Bacteroides* spp | 2 | 2 | 0 | 0 | 0 | 0 |
| *Fusobacterium necrophorum* | 1 | 1 | 0 | 0 | 0 | 0 |
| *Porphyromonas asaccharolytica* sp | 1 | 0 | 0 | 1 | 0 | 0 |
| *Prevotella nigrescens* | 1 | 0 | 0 | 0 | 0 | 1 |
| *Prevotella denticola* | 1 | 0 | 0 | 0 | 0 | 1 |
| **Others** |  |  |  |  |  |  |
| *Aeromonas caviae* | 1 | 1 | 0 | 0 | 0 | 0 |
| *Aeromonas hydrophila* | 2 | 1 | 0 | 0 | 0 | 1 |
| *Aeromonas jandaei* | 1 | 0 | 0 | 0 | 0 | 1 |
| *Aeromonas* spp | 2 | 0 | 0 | 0 | 0 | 2 |
| *Pasteurella bettyae* | 1 | 0 | 0 | 1 | 0 | 0 |
| *Pasteurella canis* | 1 | 1 | 0 | 0 | 0 | 0 |
| *Pasteurella multocida* | 3 | 2 | 0 | 0 | 1 | 0 |
| *Pasteurella* spp | 1 | 1 | 0 | 0 | 0 | 0 |
| **Gram-positive cocci** |  |  |  |  |  |  |
| **Staphylococci** |  |  |  |  |  |  |
| *Staphylococcus aureus* | 132 | 36 | 9 | 11 | 24 | 52 |
| *Staphylococcus capitis* | 1 | 0 | 0 | 0 | 1 | 0 |
| *Staphylococcus caprae* | 4 | 0 | 0 | 0 | 3 | 1 |
| *Staphylococcus cohnii* | 1 | 0 | 0 | 0 | 0 | 1 |
| *Staphylococcus epidermidis* | 16 | 1 | 0 | 2 | 10 | 3 |
| *Staphylococcus haemolyticus* | 1 | 0 | 0 | 0 | 0 | 1 |
| *Staphylococcus lugdunensis* | 4 | 1 | 0 | 0 | 1 | 2 |
| *Staphylococcus simulans* | 1 | 0 | 0 | 0 | 0 | 1 |
| **Streptococci** |  |  |  |  |  |  |
| *Streptococcus acidominimus* | 1 | 1 | 0 | 0 | 0 | 0 |
| *Streptococcus agalactiae* | 24 | 10 | 4 | 0 | 6 | 4 |
| *Streptococcus anginosus* | 7 | 3 | 0 | 1 | 1 | 2 |
| *Streptococcus constellatus* | 4 | 1 | 0 | 2 | 0 | 1 |
| *Streptococcus dysgalactiae* | 7 | 5 | 0 | 0 | 1 | 1 |
| *Streptococcus equinus* | 1 | 1 | 0 | 0 | 0 | 0 |
| *Streptococcus gallolyticus* | 4 | 0 | 2 | 0 | 2 | 0 |
| *Streptococcus gordonii* | 1 | 1 | 0 | 0 | 0 | 0 |
| *Streptococcus intermedius* | 1 | 1 | 0 | 0 | 0 | 0 |
| *Streptococcus mitis* | 7 | 3 | 1 | 0 | 1 | 2 |
| *Streptococcus pneumoniae* | 5 | 5 | 0 | 0 | 0 | 0 |
| *Streptococcus pyogenes* | 8 | 4 | 0 | 0 | 1 | 3 |
| *Streptococcus suis* | 1 | 1 | 0 | 0 | 0 | 0 |
| **Enterococci** |  |  |  |  |  |  |
| *Enterococcus avium* | 2 | 0 | 0 | 0 | 0 | 2 |
| *Enterococcus faecalis* | 7 | 1 | 1 | 0 | 0 | 5 |
| *Enterococcus faecium* | 2 | 1 | 0 | 0 | 0 | 1 |
| *Enterococcus raffinosus* | 1 | 0 | 0 | 0 | 0 | 1 |
| **Anaerobes** |  |  |  |  |  |  |
| *Finegoldia magna* | 3 | 0 | 0 | 0 | 0 | 3 |
| *Parvimonas micra* | 1 | 0 | 0 | 0 | 0 | 1 |
| *Peptostreptococcus anaerobius* | 1 | 0 | 0 | 0 | 0 | 1 |
| **Others** |  |  |  |  |  |  |
| *Abiotrophia defectiva* | 1 | 0 | 0 | 0 | 1 | 0 |
| **Gram-positive bacilli** |  |  |  |  |  |  |
| **Aerobes** |  |  |  |  |  |  |
| *Bacillus cereus* | 3 | 0 | 0 | 0 | 1 | 2 |
| *Corynebacterium amycolatum* | 2 | 0 | 0 | 1 | 0 | 1 |
| *Trueperella bernardiae* | 1 | 0 | 0 | 1 | 0 | 0 |
| *Nocardia cyriacigeorgica* | 1 | 0 | 0 | 0 | 0 | 1 |
| **Anaerobes** |  |  |  |  |  |  |
| *Clostridium glycolicum* | 1 | 0 | 0 | 0 | 0 | 1 |
| *Cutibacterium acnes* | 5 | 0 | 1 | 0 | 2 | 2 |
| **Gram-negative cocci** |  |  |  |  |  |  |
| *Neisseria flava* | 1 | 1 | 0 | 0 | 0 | 0 |
| *Neisseria gonorrhoeae* | 1 | 1 | 0 | 0 | 0 | 0 |
| **Mycobacteria** |  |  |  |  |  |  |
| *Mycobacterium genavense* | 1 | 1 | 0 | 0 | 0 | 0 |
| *Mycobacterium tuberculosis* | 1 | 0 | 1 | 0 | 0 | 0 |
| **Fungi** |  |  |  |  |  |  |
| *Candida parapsilosis* | 1 | 0 | 0 | 0 | 0 | 1 |
| HACEK= *Haemophilus* spp., *Aggregatibacter* spp., *Cardiobacterium hominis*, *Eikenella corrodens*, and *Kingella* spp.; a group of fastidious Gram-negative bacilli that are part of the normal oral and upper respiratory tract flora.  sp=species; spp=species pluralis. | | | | | | |

Table *1* – Baseline characteristics

^a^Ten patients presented with two BJI conditions and, to avoid duplicates in data analysis, were included in only a single subgroup after medical history review (details: one patient with septic knee arthritis and femoral osteomyelitis was analyzed as septic arthritis; two with prosthetic hip infection and secondary spondylodiscitis as a prosthetic joint infection; two with septic hip arthritis and iliac osteomyelitis as septic arthritis; two with osteosynthesis-associated bone infection above a prosthesis as osteosynthesis-associated bone infections; and 4 with osteoarticular infection of a finger or toe as osteomyelitis).

^b^Osteomyelitis encompasses cases resulting from hematogenous spread (n=2), contiguous extension (n=14), direct inoculation (n=3) or unknown (n=1), as well as septic pseudarthrosis (n=3).

^c^Native septic arthritis most affected the knee (48/88, 55%), hand (10/88, 12%) and hip (7/88, 8%). Spondylodiscitis was located in the cervical (n=2/27, 7%), thoracic (n=9/27, 33%), lumbar (n=15/27, 56%) or multiples spinal regions (n=1/27, 4%). Osteomyelitis most affected the hand (9/23, 39%), femur (5/23, 22%), and both the leg and foot (4/23, 17% each). Prosthetic joint infections most affected the knee (36/71, 51%), hip (34/71, 48%) and shoulder (1/71, 1%). Most cases involved total joint arthroplasties (55/71, 77%), followed by hemiarthroplasties (14/71, 20%), and spacers (2/71, 3%). Osteosynthesis-associated infections involved most affected the femur (25/103, 24%), tibia or fibula (22/103, 21%), and ankle (17/103, 17%). The hardwares involved were screws (44/103, 43%), plates (40/103, 39%), Kirschner wires (21/103, 20%), intramedullary nails (18/103, 17%), external fixators (18/103, 17%), cerclage wire (6/103, 6%), intramedullary Kirschner wires, and endobuttons (1/103, 1% each).

Figure 1 - Proportion of patients (%) with at least one isolate from each microbial group, stratified by type of bone and joint infection (BJI).

Among all BJI, Gram-negative bacilli (GNB) accounted for 39% of isolates (123/312), including AmpC β-lactamase–producing Enterobacterales (16%, 50/312) and *Pseudomonas aeruginosa* (12%, 38/312), while *Staphylococcus aureus* was the most commonly isolated bacterium (42%, 132/312). Among the streptococcal species (21%, 66/312), *Streptococcus agalactiae* was the most frequently isolated species (8%, 24/312). Coagulase-negative staphylococci (CoNS) (7%, 23/312), anaerobes (5%, 16/312), and enterococci (3%, 10/312) were less frequently detected. *Pasteurella* spp. and *Aeromonas* spp. were also identified (2% each, 6/312). In native septic arthritis, GNB accounted for 31% of isolates (27/88), including AmpC β-lactamase–producing Enterobacterales (10%, 9/88) and *Pseudomonas aeruginosa* (9%, 8/88), while *Staphylococcus aureus* was the most commonly isolated bacterium (41%, 36/88). Among the streptococcal species (36%, 32/88), *Streptococcus agalactiae* was the most frequently isolated (11%, 10/88). In spondylodiscitis, GNB were identified in nearly one-third of infections (33%, 9/27), with a predominance of *Escherichia coli* (19%, 5/27). Streptococci accounted for 26% of isolates (7/27), including *Streptococcus agalactiae* in four cases (15%, 4/27). Among osteomyelitis cases, GNB were involved in 52% of patients (12/23), with no predominant species, followed by *Staphylococcus aureus* (48%, 11/23). In prosthetic joint infections, GNB accounted for 38% of isolates (27/71), including *Pseudomonas aeruginosa* (11%, 8/71), AmpC β-lactamase–producing Enterobacterales (8%, 6/71), and *Klebsiella pneumoniae* (8%, 6/71). *Staphylococcus aureus* and CoNS were identified in 34% (24/71) and 20% of patients (14/71), respectively, while streptococci were isolated in 17% of cases (12/71), including *Streptococcus agalactiae* (8%, 6/71). Anaerobic bacteria were present in 4% (3/71) of patients, including *Cutibacterium acnes* (3%, 2/71). Methicillin-resistant strains were observed in 9% (6/71) of patients, while ESBL-producing Enterobacterales were detected in 6% (4/71) of patients. In osteosynthesis-associated infections, GNB were involved in 47% (48/103) of patients, with AmpC β-lactamase–producing Enterobacterales identified in 31% (32/103) of patients and *Pseudomonas aeruginosa* in 19% (19/103) of patients. *Staphylococcus aureus* and CoNS were identified in 51% (52/103) and 5% (5/103) of patients, respectively, while streptococci were isolated in 12/103 (12%) cases, including *Streptococcus agalactiae* (4%, 4/103). Enterococcal species were identified in 8% (8/103) of patients. Anaerobic bacteria were present in 8% (8/103) of patients, including *Cutibacterium acnes* (2%, 2/103). Methicillin-resistant strains were observed in 5% (5/103) of patients, while ESBL-producing Enterobacterales were detected in 3% (3/103) of patients.
